# Evaluation of multi-task learning in deep learning-based positioning classification of mandibular third molars

**DOI:** 10.1038/s41598-021-04603-y

**Published:** 2022-01-13

**Authors:** Shintaro Sukegawa, Tamamo Matsuyama, Futa Tanaka, Takeshi Hara, Kazumasa Yoshii, Katsusuke Yamashita, Keisuke Nakano, Kiyofumi Takabatake, Hotaka Kawai, Hitoshi Nagatsuka, Yoshihiko Furuki

**Affiliations:** 1 Department of Oral and Maxillofacial Surgery, Kagawa Prefectural Central Hospital, 1-2-1, Asahi-machi, Takamatsu, Kagawa 760-8557 Japan; 2 Department of Oral Pathology and Medicine, Okayama University Graduate School of Medicine, Dentistry and Pharmaceutical Sciences, 2-5-1 Shikatacho, Kita-ku, Okayama, 700-8525 Japan; 3 Department of Molecular Oral Medicine and Maxillofacial Surgery, Graduate School of Biomedical and Health Sciences, Hiroshima University, 1-2-3 Kasumi, Minami-ku, Hiroshima, 734-8553 Japan; 4 Department of Electrical, Electronic and Computer Engineering, Faculty of Engineering, Gifu University, 1-1 Yanagido, Gifu, Gifu 501-1193 Japan; 5 Center for Healthcare Information Technology, Tokai National Higher Education and Research System, 1-1 Yanagido, Gifu, Gifu 501-1193 Japan; 6 Department of Intelligence Science and Engineering, Graduate School of Natural Science and Technology, Gifu University, 1-1 Yanagido, Gifu, Gifu 501-1193 Japan; 7 Polytechnic Center Kagawa, 2-4-3, Hananomiya-cho, Takamatsu, Kagawa 761-8063 Japan

**Keywords:** Digital radiography in dentistry, Panoramic radiography

## Abstract

Pell and Gregory, and Winter’s classifications are frequently implemented to classify the mandibular third molars and are crucial for safe tooth extraction. This study aimed to evaluate the classification accuracy of convolutional neural network (CNN) deep learning models using cropped panoramic radiographs based on these classifications. We compared the diagnostic accuracy of single-task and multi-task learning after labeling 1330 images of mandibular third molars from digital radiographs taken at the Department of Oral and Maxillofacial Surgery at a general hospital (2014–2021). The mandibular third molar classifications were analyzed using a VGG 16 model of a CNN. We statistically evaluated performance metrics [accuracy, precision, recall, F1 score, and area under the curve (AUC)] for each prediction. We found that single-task learning was superior to multi-task learning (all p < 0.05) for all metrics, with large effect sizes and low p-values. Recall and F1 scores for position classification showed medium effect sizes in single and multi-task learning. To our knowledge, this is the first deep learning study to examine single-task and multi-task learning for the classification of mandibular third molars. Our results demonstrated the efficacy of implementing Pell and Gregory, and Winter’s classifications for specific respective tasks.

## Introduction

The mandibular third molar is one of the most commonly impacted teeth. Treatment requires tooth extraction surgery, and extraction of the third molar is one of the most common surgical procedures worldwide. Since mandibular third molars cause various complications, surgical treatment is primarily performed to treat the symptoms associated with impaction^1,2^ and prevent conditions that impair oral health, such as future dentition malocclusion^3^. Infection and neuropathy are common complications that occur after extraction of the mandibular third molars; it is known that the position of these molars influences the occurrence of postoperative complications^4,5^. Therefore, an accurate understanding of the position of the mandibular third molars based on preoperative radiographs taken before surgery leads to safer treatment.

Pell and Gregory^6^, and Winter’s classifications^7^ are often used for classifying third molars. In the Pell and Gregory classification, the mandibular third molars are classified according to their position with respect to the second molars and the ramus of the mandible; in addition, the position of the mandibular third molar in the mesio-distal relationship is classified into classes I, II, and III, and the part of the mandibular third molar in depth is classified into levels A, B, and C. Based on the Winter’s classification, the slope category is classified with respect to the vertical axis of the mandibular third molar. These classifications help describe the condition of the third molar of the lower jaw among dentists using a standardized language and make it easier to understand the difficulty of tooth extraction. In addition, diagnosis using these classifications is effective not only for sharing diagnosis information before tooth extraction but also for feedback after tooth extraction; additionally, these classifications are important from an educational perspective.

Deep learning is a machine learning method that can automatically detect the functions required to predict a specific result from the given data. Complex learning is possible using a deep convolutional neural network (CNN) with multiple layers between inputs and outputs. Many achievements have been made in the application of these technologies in the medical field. In particular, analyses using deep learning based on medical images have provided comprehensive knowledge because this methodology can interpret data complexity more appropriately than standard statistical methods. In the field of dentistry, this methodology has also been applied to the identification and diagnosis of dental caries^8^, endodontic lesions^9^, dental implants^10^, orthodontic diagnoses^11^, and osteoporosis^12^. Various methods are currently being developed for use in machine learning. Among these, the multi-task learning method learns multiple classification items simultaneously, enabling multiple predictive diagnoses^13^. This efficient machine learning method may improve performance compared to single-task learning by evaluating interrelated concepts.

This study aimed to present a CNN-based deep learning model using panoramic radiographs according to Pell and Gregory, and Winter’s classifications, with the purpose of locating the precise positioning of the mandibular third molars. Furthermore, we propose multi-task learning as another approach for analyzing medical images while improving the generalization function of multiple tasks. In addition, we aimed to evaluate the accuracy of position classification of the mandibular third molars via multi-task deep learning.

## Results

### Prediction performance

#### Performance of the single-task model

Performance metrics for each of the single-task model are shown in Table 1. Position classification showed high performance metrics in a single-task. Supplementary Fig. S1 shows the ROC curves of single-task learning at tenfold.

**Table 1:** Prediction performance on the single-task model.

	Accuracy	Precision	Recall	F1 score	AUC
	SD	SD	SD	SD	SD
	95%CI	95%CI	95%CI	95%CI	95%CI
Class	0.8541	0.8588	0.8544	0.8538	0.9638
	0.0074	0.0075	0.0071	0.0073	0.0018
	0.851–0.858	0.856–0.862	0.852–0.857	0.851–0.857	0.963–0.965
Position	0.8895	0.8824	0.8877	0.8831	0.9739
	0.0055	0.0075	0.0064	0.0064	0.0017
	0.887–0.892	0.880–0.885	0.885–0.890	0.881–0.886	0.973–0.975
Winter's classification	0.8663	0.8559	0.8003	0.8138	0.9801
	0.0052	0.0143	0.0119	0.0123	0.0025
	0.864–0.868	0.851–0.861	0.796–0.805	0.809–0.818	0.979–0.981

*SD* standard deviation, *95% CI* 95% confidence interval, *AUC* area under the receiver operating characteristics curve.

#### Performance of the multi-task model

Table 2 shows the performance metrics of the three-task multi-task model, including information on class, position, and Winter's classification. Table 3 shows the performance metrics for the two-task multi-task model, including information on class and position. Supplementary Fig. S1 shows the ROC curves of the two-type multi-task learning at tenfold.

**Table 2:** Prediction performance of the multi-task model, including class, position and Winter’s classification.

	Accuracy	Precision	Recall	F1 score	AUC
	SD	SD	SD	SD	SD
	95%CI	95%CI	95%CI	95%CI	95%CI
Class	0.8487	0.8541	0.8478	0.8474	0.9606
	0.0087	0.0065	0.0083	0.0084	0.0018
	0.845–0.852	0.851–0.857	0.845–0.851	0.844–0.851	0.960–0.961
Position	0.8861	0.8779	0.8829	0.8781	0.9733
	0.0056	0.0065	0.0084	0.0070	0.0025
	0.884–0.888	0.875–0.880	0.880–0.886	0.875–0.881	0.972–0.974
Winter's classification	0.8537	0.8332	0.7747	0.7896	0.9770
	0.0068	0.0124	0.0105	0.0110	0.0024
	0.851–0.856	0.829–0.861	0.771–0.779	0.786–0.793	0.976–0.978

*SD* standard deviation, *95% CI* 95% confidence interval, *AUC* area under the receiver operating characteristics curve.

**Table 3:** Prediction performance of the two-task multi-task model including class and position.

	Accuracy	Precision	Recall	F1 score	AUC
	SD	SD	SD	SD	SD
	95%CI	95%CI	95%CI	95%CI	95%CI
Class	0.8543	0.8590	0.8539	0.8534	0.9633
	0.0094	0.0102	0.0094	0.0088	0.0028
	0.887–0.892	0.856–0.862	0.850–0.857	0.850–0.857	0.962–0.964
Position	0.8899	0.8814	0.8857	0.8813	0.9737
	0.0069	0.0102	0.0077	0.8813	0.0018
	0.772–0.814	0.878–0.885	0.882–0.891	0.878–0.884	0.973–0.974

*SD* standard deviation, *95% CI* 95% confidence interval, *AUC* area under the receiver operating characteristics curve.

#### Comparison of the single-task and multi-task models in terms of performance metrics

Table 4 shows the statistical evaluation results of the single- and multi-task models for each performance metric. Comparing the two groups by p-value, the single-task model was superior to the multi-3task model, and the single-task model was superior to the standard statistical approach for all metrics. In the single-task and multi-2task (class and position) models, the single-task model was superior in all metrics except the AUC for position classification.

**Table 4:** Statistical comparisons by p-value and effect size for the single-task and multi-task models.

Class	Accuracy	Precision	Recall	F1 score	AUC
**P value**					
Multi3	0.029	0.064	0.006	0.006	< 0.0001
Multi2	0.996	0.994	0.955	0.966	0.523
**Effect size**					
Multi3	0.674	0.554	0.857	0.817	1.823
Multi2	0.019	0.0235	0.064	0.057	0.233

Position	Accuracy	Precision	Recall	F1 score	AUC
**P value**					
Multi3	0.056	0.062	0.030	0.014	0.447
Multi2	0.947	0.851	0.491	0.497	0.887
**Effect size**					
Multi3	0.616	0.651	0.641	0.759	0.267
Multi2	0.068	0.1122	0.281	0.258	0.122

Winter's classification	Accuracy	Precision	Recall	F1 score	AUC
**P value**					
Multi3	< 0.0001	< 0.0001	< 0.0001	< 0.0001	< 0.0001
**Effect size**					
Multi3	2.082	1.699	2.285	2.072	1.238

*SD* standard deviation, *95% CI* 95% confidence interval, *AUC* area under the receiver operating characteristics curve.

Regarding effect size, in the single-task and multi-3task models, the effect size was large for all metrics except position classification (AUC and p-value). On the contrary, in the single-task and multi-2task models, recall and F1 score (in the position classification) showed medium effect sizes, and all other parameters showed small effect sizes. The power in the post hoc analysis of this study was 1.0 for all performance metrics.

### Visualization

**Figure 1:**
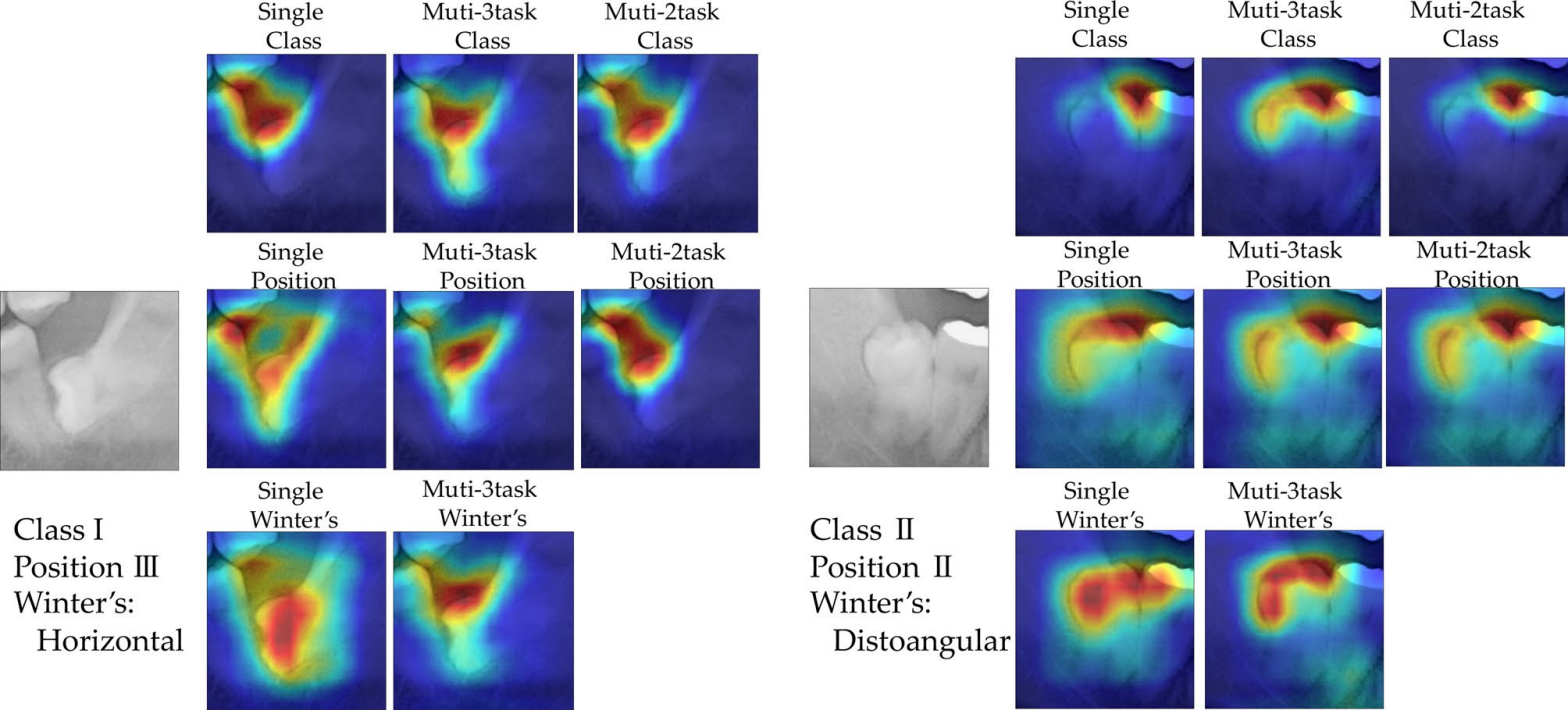
Visualization of the judgment basis for classification prediction by a convolutional neural network (CNN) using Grad-CAM.

Grad-CAM was used to explain the prediction process for the CNN in terms of identifying each category. Consequently, we visualized the judgment basis for determining the identification image area used for classification (Fig. 1, Supplementary Fig. S2). For the classification of class and position, the space above the mandibular third molar is regarded as a characteristic area of the CNN judgment basis. In contrast, Winter’s classification was used as a characteristic area for classification judgment of the entire crown of the mandibular third molar. In the multi-task models, in addition to the characteristics for each task, the characteristics of other simultaneously learned tasks were added to the criteria. In addition, because of a tendency of multi-task feature areas, we mainly focused on areas that are common to these models.

## Discussion

In this deep learning study, mandibular third molar classification (class, position, Winter's classification) was performed in single-task and multi-task models. In multi-task models, wherein three classification tasks are simultaneously performed for each single-task, we found that the classification evaluation metric was statistically superior to that of the multi-task models. There was no significant difference in classification accuracy between and single-task models and the two classification multi-task model..

Multi-task modeling uses inductive transfer to improve task learning using signals from related tasks discovered during training^20^. Multi-tasks have a great advantage in reducing calculation costs because they can perform multiple tasks simultaneously. In fact, in our research, we observed a significant difference when comparing the total number of parameters for each single-task and the number of parameters for multiple tasks. In addition, multiple tasks can improve the accuracy of other classifications by learning the characteristics common to each task^13,21^. However, in our results, the classification performance of multi-task models decreased after three tasks. This may be because each task has classification criteria for different characteristics. Thus, in multi-task models, classification performance may be degraded due to conflicting areas of interest for the classification of each task.

The mandibular third molar classifications performed in this study were the Pell and Gregory classification as well as Winter’s classification. In the Pell and Gregory classification, certain classes and positions are classified according to the mesio-distal positional relationship and vertical depth of the mandibular third molar^6^. Accuracy was improved by simultaneously performing these two tasks. Unfortunately, no statistically significant improvement in performance metrics was observed. On the contrary, we found a statistically significant decrease in classification performance of the three task multi-task with the addition of Winter’s classification. Specifically, in Winter’s classification, the angulation and inclination of the mandibular third molar are judged, with the orientation of the mandibular third molar as the criterion^7^. Because feature extraction is weighted toward the entire mandibular third molar, the features for predicting CNN were possibly different from those of the Pell and Gregory classification.

A few studies have used deep learning to classify the position of the mandibular third molar. Yoo et al.^22^ performed class, position, and Winter’s classifications of the mandibular third molar. Additionally, the observed accuracy was 78.1% for class, 82.0% for position, and 90.2% for Winter's classification. Although Winter’s classification cannot be compared because all evaluations had not been performed, our results are more accurate for class and position.

For the weights learnt by the CNN, Grad-CAM can use the gradient of the classification score for convolutional features determined by the network to understand which parts of the image are most important for classification^19^. Grad-CAM can visualize the judgment basis for learning by CNN, which is regarded as a black box. In this study, visualization was performed using the gradient of the final convolution layer. Visualization results for Grad-CAM class and position classifications often show similar feature areas, while Winter’s classifications primarily assign features to the entire crown. Interestingly, in the multi-task models, the characteristics of the other tasks were added to the judgement basis together with the characteristics of each task. Therefore, the rate of classification errors may have been increased by referring to other parts that deviated from the judgment basis based on the original most notable features in multi-task.

Since statistically significant differences are easily recognized in proportion to the sample size within statistical hypothesis tests between two groups, effect sizes and statistically significant differences are important for evaluating substantial differences^23^. Effect size can be interpreted as a value that indicates the actual magnitude of the difference, which does not depend on the unit of measurement; this is one of the most important indicators for analysis. In this study, there was a correlation between the statistical hypothesis test and effect size in the two groups, and statistical evaluations showed that the sample size was appropriate. Our study is the first to show the effect size for the evaluation of mandibular third molar position classification using deep learning. The effect sizes calculated from this experiment will be useful when pre-designing the sample size in a similar study. To our knowledge, there are a few reports on the calculation of effect sizes for comparison between deep learning models.

Diagnosis of the third mandibular molar is the most common oral surgery and is important not only for oral and maxillofacial surgeons, but also for general dentists. Accurate diagnosis leads to safe tooth extraction. In the future, as an auxiliary diagnosis, it is desirable to automatically diagnose the mandibular third molar using deep learning on the captured digital panoramic X-ray image. For this purpose, we would like to work on automatic detection using object detection of the mandibular third molar.

The strength of our study over previous studies is that the influence of multi-task learning was statistically evaluated. The mandibular third molar classification grouping performed in this study was as close as possible to the clinical setting. To the best of our knowledge, this is the first study to statistically and visually reveal the influence of multi-task learning on mandibular third molar classification by deep learning. Grad-CAM revealed areas of interest for each model of CNN. Additionally, the calculated effect size can be used to estimate the sample size for future studies: it is suitable for statistically evaluating results correctly, rather than simply comparing values between different groups.

This study had several limitations. First, the amount of data for the current evaluation was modest. Especially in the Winter’s classification, there are few buccolingual and inverted results, which could result in bias. We verified our findings using a stratified K-fold CV to avoid any bias in the data set for training; however, it is important to conduct further studies with a larger amount of data. Second, the CNN type was VGG16 only. In the future, CNNs with various characteristics should be evaluated, and it will be necessary to verify the most suitable CNN. The third limitation is the search for a Pareto optimal solution. In multi-task learning, classification performance is degraded due to conflicting areas of interest for the classification of each task. Therefore, in multi-task learning, it is necessary to consider the ratio of the gradients of loss function, wherein the gradients of each task are relatively balanced.

## Conclusions

To our knowledge, this is the first deep learning study of the classification (class, position, Winter) of the mandibular third molar to examine single-task and multi-task models. The multi-task model with two tasks (class and position) was not statistically significantly different from single-task models, and the three multi-task classifications were statistically significantly less accurate than the respective single-task classifications. Finally, we found that, in the deep learning classification of the mandibular third molar, it is more effective to classify the Pell and Gregory, and Winter’s classifications based on their respective tasks. Our results will greatly contribute to the development of automatic classification and diagnosis of mandibular third molars from individual panoramic radiograph images in the future.

## Materials and methods

### Study design

The purpose of this study was to evaluate the classification accuracy of CNN-based deep learning models using cropped panoramic radiographs according to the Pell and Gregory, and Winter’s classifications for the location of the mandibular third molars. Supervised learning was chosen as the method for deep learning analysis. We compared the diagnostic accuracy of single-task and multi-task learning.

### Data acquisition

We used retrospective radiographic image data collected from April 2014 to December 2020 at a single general hospital. This study was approved by the Institutional Review Boards of the respective institutions hosting this work (the Institutional Review Boards of Kagawa Prefectural Central Hospital, approval number 1020) and was conducted in accordance with the ethical standards of the Declaration of Helsinki and its later amendments. Informed consent was waived for this retrospective study because no protected health information was used by the Institutional Review Boards of Kagawa Prefectural Central Hospital. Study data included patient’s aged 16–76 years who had panoramic radiographs taken at our hospital prior to extracting their mandibular third molars.

In the Pell and Gregory classification, the mandibular second molar was the diagnostic criterion. Therefore, cases of mandibular second molar defects, impacted teeth, and residual roots were excluded from this study. Additionally, we excluded cases of unclear images, residual plates after mandibular fracture, and residual third molar root or tooth extraction interruptions. Overall, we excluded residual third molar roots (39 teeth), mandibular second molar defects or residual teeth (15 teeth), impacted mandibular second molars (12 teeth), tooth extraction interruptions of third molars (9 teeth), unclear images (3 teeth), and residual plates after mandibular fracture (1 tooth). In total, 1,330 mandibular third molars were retained for further deep learning analysis.

### Data preprocessing

**Figure 2:**
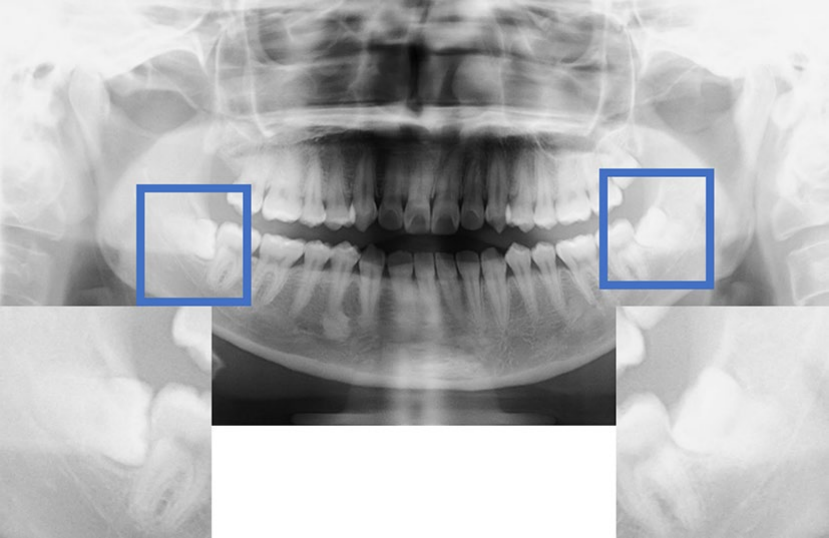
A depiction of the crop method for data preprocessing.

Images were acquired using dental digital panoramic radiographs (AZ3000CMR or Hyper-G CMF, Asahiroentgen Ind. Co., Ltd., Kyoto, Japan). All digital image data were output in Tagged Image File Format format (2964 × 1464, 2694 × 1450, 2776 × 1450, or 2804 × 1450 pixels) via the Kagawa Prefectural Central Hospital Picture Archiving and Communication Systems system (Hope Dr Able-GX, Fujitsu Co., Tokyo, Japan). Two maxillofacial surgeons manually identified areas of interest on the digital panoramic radiographs using Photoshop Elements (Adobe Systems, Inc., San Jose, CA, USA) under the supervision of an expert oral and maxillofacial surgeon. The method of cropping the image was to cut out the mandibular second molar and the ramus of the mandible in the mesio-distal direction and completely include the apex of the mandibular third molar in the vertical direction (Fig. 2). The cropped images had a resolution of 96 dpi/inch, and each cropped image was saved in portable network graphics format.

The manual method of cropping the image involved cutting out the mandibular second molar and the ramus of the mandible in the mesio-distal direction as well as completely including the apex of the mandibular third molar in the vertical direction.

### Classification methods

Pell and Gregory classification^6^ is categorized into class and position components. The classification was performed according to the positional relationship between the ramus of the mandible and the mandibular second molar in the mesio-distal direction. The distribution of the mandibular third molar classification is shown in Table 5.

**Table 5:** Distribution of Pell and Gregory, and Winter’s classifications.

Pell & Gregory classification				Winter's classification	
Class		Position			
I	405	A	438	Horizontal	514
				Mesioangular	346
II	607	B	693	Vertical	282
				Distoangular	79
III	318	C	199	Inverted	79
				Bucco/lingualangular	30

Class I: The distance from the distal surface of the second molar to the anterior margin of the mandibular ramus was larger than the diameter of the third molar crown.

Class II: The distance from the distal surface of the second molar to the anterior margin of the mandibular ramus was smaller than the diameter of the third molar crown.

Class III: Most third molars are present in the ramus of the mandible. Position classification was performed according to the depth of the mandibular second molar.

Level A: The occlusal plane of the third molar was at the same level as the occlusal plane of the second molar.

Level B: The occlusal plane of the third molar is located between the occlusal plane and the cervical margin of the second molar.

Level C: The third molar was below the cervical margin of the second molar.

Based on Winter’s classification, the mandibular third molar is classified into the following six categories^7,14^:

Horizontal: The long axis of the third molar is horizontal (from 80° to 100°).

Mesioangular: The third molar is tilted toward the second molar in the mesial direction (from 11° to 79°).

Vertical: The long axis of the third molar is parallel to the long axis of the second molar (from 10° to –10°).

Distoangular: The long axis of the third molar is angled distally and posteriorly away from the second molar (from − 11° to − 79°).

Inverted: The long axis of the third molar is angled distally and posteriorly away from the second molar (from 101° to –80°).

Buccoangular or lingualangular: The impacted tooth is tilted toward the buccal-lingual direction.

### CNN model architecture

The study evaluation was performed using the standard deep CNN model (VGG16) proposed by the Oxford University VGG team^15^. We performed a normal CNN consisting of a convolutional layer and a pooling layer for a total of 16 layers of weight (i.e., convolutional and fully connected layers).

With efficient model construction, fine-tuning the weight of existing models as initial values for additional learning is possible. Therefore, the VGG 16 model was used to transfer learning with fine-tuning, using pre-trained weights in the ImageNet database^16^. The process of deep learning classification was implemented using Python (version 3.7.10) and Keras (version 2.4.3).

### Data set and model training

The model training was generalized using K-fold cross-validation in the model training algorithm. Our deep learning models were evaluated using tenfold cross-validation to avoid overfitting and bias and to minimize generalization errors. The dataset was split into ten random subsets using stratified sampling to retain the same class distribution across all subsets. Within each fold, the dataset was split into separate training and test datasets using a 90% to 10% split. The model was trained 10 times to obtain the prediction results for the entire dataset, with each iteration holding a different subset for validation. Data augmentation can be found in the appendix.

### Multi-task

**Figure 3:**
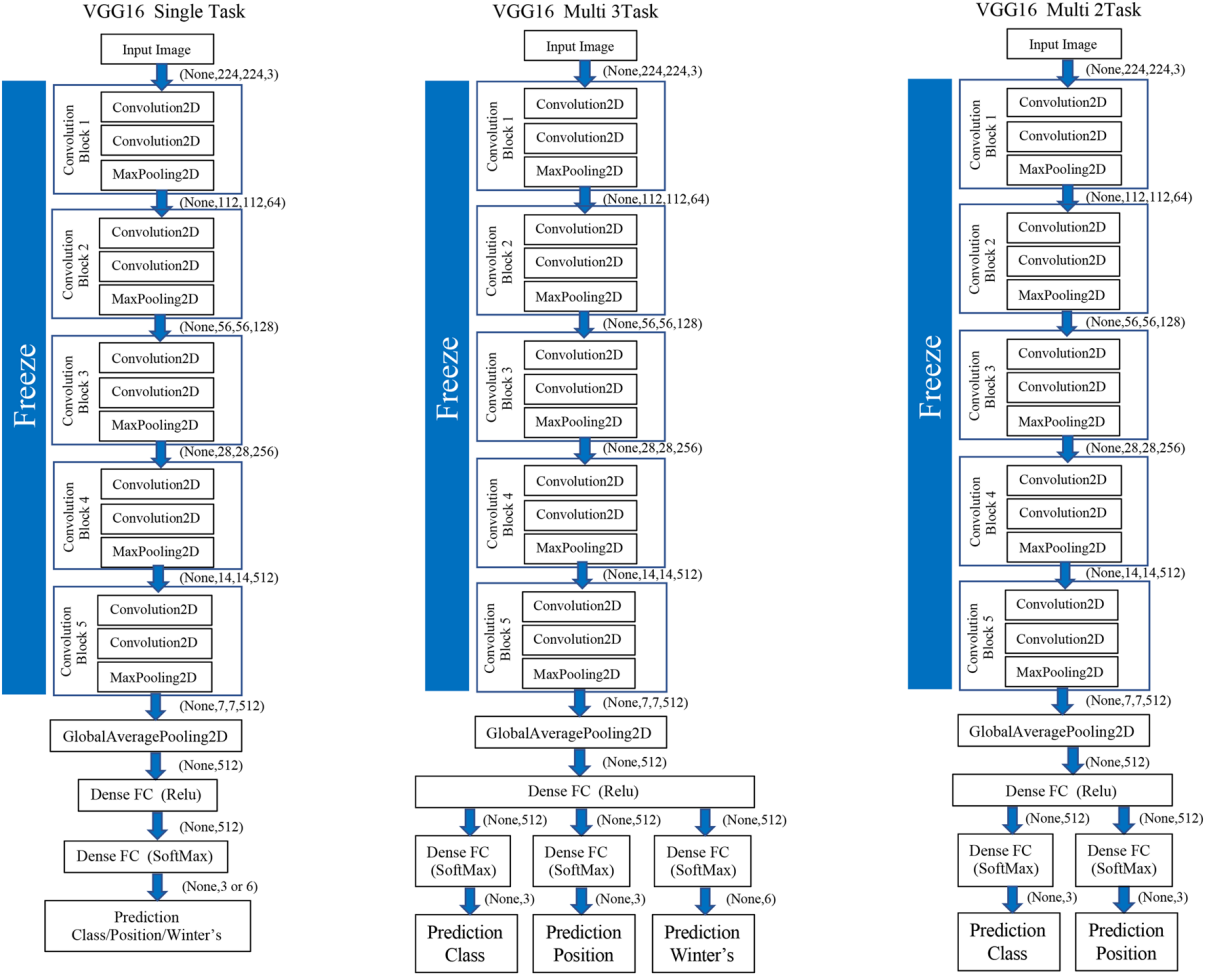
Schematic diagram for classification of the mandibular third molars using single-task and multi-task convolutional neural network (CNN) models.

As another approach to the mandibular third molar classifier, a deep neural network with multiple independent outputs was implemented and evaluated. There are two proposed multi-task CNNs. One is a CNN model that can analyze the three tasks of the Pell and Gregory, and Winter’s classifications simultaneously. The other is a CNN model that can simultaneously analyze the class and position classifications that constitute the Pell and Gregory classification. These models can significantly reduce the number of trainable parameters required when using two or three independent CNN models for mandibular third molar classification. The proposed model has a feature learning shared layer that includes a convolutional layer and a max-pooling layer that are shared with two or three separate branches and independent, fully connected layers used for classification. For the classification, two or three separate branches consisting of dense layers were connected to each output layer of the Pell and Gregory, and Winter’s classifications. Each branch included softmax activation. (Fig. 3) Table 6 shows the number of parameters for each of the two types of multi-tasks and single-tasks in the VGG 16 model.

**Table 6:** The number of parameters for each of the two types of multi-tasks and single tasks in the VGG16 model.

VGG16	Total parameter	Trainable parameter	Non-trainable parameter
Multi-3task (class, position, and Winter's)	**15,252,307**	**537,612**	**14,714,695**
Multi-2task (class and position) + Single-task (Winter's)	**30,492,314**	**1,062,924**	**29,429,390**
Multi-2task (class and position)	15,246,157	531,462	14,714,695
Single-task (Winter's)	15,243,082	528,387	14,714,695
Single-task (class + position + Winter's)	**45,729,246**	**1,585,161**	**44,144,085**
Each single-task (class/position/Winter's)	15,243,082	528,387	14,714,695

Bold is the sum of the parameters for each task.

In the multi-task model, each model was implemented to learn the classification of the mandibular third molars. In both training, the cross entropy calculated in (Eq. 1) was used as the error function. The total error function (L_3total) of the multi-task model for the three proposed tasks is the sum of the Pell and Gregory classification class and position prediction errors (L_cls), (L_pos), and Winter’s classification prediction errors (L_wit) (Eq. 2):

1 $$L=-\sum_{i=0}{t}_{i}\mathit{log}{y}_{i} (a) ({t}_{i}:{\text{ correct data}}, {y}_{i}:{\text{ predicted probability of class i}})$$

2 $${L}_{3total}={L}_{cls}+{L}_{pos}+{L}_{wit}$$

The error function (L_2total) of the entire multi-task model for the two tasks was the total of the prediction errors (L_cls) and (L_pos) of class and position, as well as the Winter’s classification (Eq. 3):

3 $${L}_{2total}={L}_{cls}+{L}_{pos}$$

### Deep learning procedure

All CNN models were trained and evaluated on a 64-bit Ubuntu 16.04.5 LTS operating system with 8 GB of memory and an NVIDIA GeForce GTX 1080 (8 GB graphics processing unit). The optimizer used stochastic gradient descent with a fixed learning rate of 0.001 and a momentum of 0.9, which achieved the lowest loss on the validation dataset after multiple experiments. The model with the lowest loss in the validation dataset was chosen for inference on the test datasets. Training was performed for 300 epochs with a mini-batch size of 32. The model was trained 10 times in the tenfold cross-validation test, and the result of the entire dataset was obtained as one set. This process was repeated 30 times for each single-task model (for class, position, Winter’s classification), multi-task model (for class and position classification [two tasks], and all three multi-tasks) using different random seeds.

### Performance metrics and statistical analysis

We evaluated the performance metrics with precision, recall, and F1 score along with the receiver operating characteristic curve (ROC) and the area under the ROC curve (AUC). The ROC curves were shown for the complete dataset from the tenfold cross-validation, producing the median AUC value. Details on the performance metrics are provided in the Appendix.

The differences between performance metrics were tested using the JMP statistical software package (https://www.jmp.com/ja_jp/home.html, version 14.2.0) for Macintosh (SAS Institute Inc., Cary, NC, USA). Statistical tests were two-sided, and p values < 0.05 were considered statistically significant. Parametric tests were performed based on the results of the Shapiro–Wilk test. For multiple comparisons, Dunnett's test was performed with single-task as a control.

Differences between each multi-task model and the single-task model were calculated for each performance metric using the Wilcoxon test. Effect sizes were calculated as Hedges' g (unbiased Cohen's d) using the following formula^17^:

$$Hedges' g = \frac{{|M}_{1}-{M}_{2}|}{s}$$

$$s=\sqrt{\frac{{(n}_{1}-1){s}_{1}^{2}+({n}_{2}-1){s}_{2}^{2}}{{n}_{1}+{n}_{2}-2}}$$

M1 and M2 are the means for the multi-task and single-task models, respectively; s1 and s2 are the standard deviations for the multi-task and single-task models, respectively, and n1 and n2 are the numbers for the multi-task and single-task models, respectively.

The effect size was determined based on the criteria proposed by Cohen et al.^18^, such that 0.8 was considered a large effect, 0.5 was considered a moderate effect, and 0.2 was considered a small effect.

### Visualization for the CNN model

CNN model visualization helps clarify the most relevant features used for each classification. For added transparency and visualization, this work used the gradient-weighted class activation maps (Grad-CAM) algorithm, which functions by capturing a specific class's vital features from the last convolutional layer of the CNN model to localize its important areas^19^. Image map visualizations are heatmaps of the gradients, with “hotter” colors representing the regions of greater importance for classification.

## Supplementary Information

Supplementary Information.

## References

[1] Moghimi M, Baart JA, Hakki Karagozoglu K, Forouzanfar T (2013). Spread of odontogenic infections: A retrospective analysis and review of the literature. Quintessence Int..

[2] Sukegawa S, Saika M, Kanno T, Nakano K, Takabatake K, Kawai H, Nagatsuka H, Furuki Y (2019). Do the presence of mandibular third molar and the occlusal support affect the occurrence and the mode of mandibular condylar fractures?. J. Hard Tissue Biol..

[3] Stanaitytė R, Trakinienė G, Gervickas A (2014). Do wisdom teeth induce lower anterior teeth crowding? A systematic literature review. Stomatologija..

[4] Sukegawa S, Yokota K, Kanno T, Manabe Y, Sukegawa-Takahashi Y, Masui M, Furuki Y (2019). What are the risk factors for postoperative infections of third molar extraction surgery: A retrospective clinical study?. Med. Oral Patol. Oral Cir. Bucal..

[5] Kang F, Sah MK, Fei G (2020). Determining the risk relationship associated with inferior alveolar nerve injury following removal of mandibular third molar teeth: A systematic review. J. Stomatol. Oral Maxillofac. Surg..

[6] Pell JG, Gregory GT (1933). Impacted mandibular third molars: Classification and modified techniques for removal. Dent. Dig..

[7] Winter, G. B. *Principles of Exodontia as Applied to the Impacted Mandibular Third Molar : A Complete Treatise on the Operative Technic with Clinical Diagnoses and Radiographic Interpretations* (American Medical Books, 1926).

[8] Khanagar SB, Al-ehaideb A, Maganur PC, Vishwanathaiah S, Patil S, Baeshen HA, Sarode SC, Bhandi S (2021). Developments, application, and performance of artificial intelligence in dentistry—A systematic review. J. Dent. Sci..

[9] Ekert T, Krois J, Meinhold L, Elhennawy K, Emara R, Golla T, Schwendicke F (2019). Deep learning for the radiographic detection of apical lesions. J. Endod..

[10] Sukegawa S, Yoshii K, Hara T, Yamashita K, Nakano K, Yamamoto N, Nagatsuka H, Furuki Y (2020). Deep neural networks for dental implant system classification. Biomolecules.

[11] Khanagar SB, Al-Ehaideb A, Vishwanathaiah S, Maganur PC, Patil S, Naik S, Baeshen HA, Sarode SS (2021). Scope and performance of artificial intelligence technology in orthodontic diagnosis, treatment planning, and clinical decision-making—A systematic review. J. Dent. Sci..

[12] Lee K-S, Jung S-K, Ryu J-J, Shin S-W, Choi J (2020). Evaluation of transfer learning with deep convolutional neural networks for screening osteoporosis in dental panoramic radiographs. J. Clin. Med..

[13] Sukegawa S, Yoshii K, Hara T, Matsuyama T, Yamashita K, Nakano K, Takabatake K, Kawai H, Nagatsuka H, Furuki Y (2021). Multi-task deep learning model for classification of dental implant brand and treatment stage using dental panoramic radiograph images. Biomolecules.

[14] Yilmaz S, Adisen MZ, Misirlioglu M, Yorubulut S (2016). Assessment of third molar impaction pattern and associated clinical symptoms in a Central Anatolian Turkish population. Med. Princ. Pract..

[15] Simonyan, K., & Zisserman, A. Very deep convolutional networks for large-scale image recognition. In *Proceedings of the 3rd International Conference on Learning Representations, ICLR 2015—Conference Track Proceedings, International Conference on Learning Representations* (ICLR, 2015).

[16] Russakovsky O, Deng J, Su H, Krause J, Satheesh S, Ma S, Huang Z, Karpathy A, Khosla A, Bernstein M, Berg AC, Fei-Fei L (2015). ImageNet large scale visual recognition challenge. Int. J. Comput. Vis..

[17] Nakagawa S, Cuthill IC (2007). Effect size, confidence interval and statistical significance: A practical guide for biologists. Biol. Rev. Camb. Philos. Soc..

[18] Cohen J (2013). Statistical Power Analysis for the Behavioral Sciences.

[19] Selvaraju RR, Cogswell M, Das A, Vedantam R, Parikh D, Batra D (2016). Grad-CAM: Visual explanations from deep networks via gradient-based localization. Int. J. Comput. Vis..

[20] Crichton G, Pyysalo S, Chiu B, Korhonen A (2017). A neural network multi-task learning approach to biomedical named entity recognition. BMC Bioinform..

[21] Zhou Y, Chen H, Li Y, Liu Q, Xu X, Wang S, Yap PT, Shen D (2021). Multi-task learning for segmentation and classification of tumors in 3D automated breast ultrasound images. Med. Image Anal..

[22] Yoo JH, Yeom HG, Shin WS, Yun JP, Lee JH, Jeong SH, Lim HJ, Lee J, Kim BC (2021). Deep learning based prediction of extraction difficulty for mandibular third molars. Sci. Rep..

[23] Greenland S, Senn SJ, Rothman KJ, Carlin JB, Poole C, Goodman SN, Altman DG (2016). Statistical tests, P values, confidence intervals, and power: a guide to misinterpretations. Eur. J. Epidemiol..

