# Supplementary material for: Evaluation of multi-task learning in deep learning-based positioning classification of mandibular third molars
Source: Sci Rep. 2022 Jan 13;12:684. doi: 10.1038/s41598-021-04603-y (PMC8758752; doi:10.1038/s41598-021-04603-y)

A deep learning-based positioning classification of the mandibular third molars: Is multi-task deep learning useful?

Shintaro Sukegawa ^1,2, *^, Tamamo Matsuyama ^3^, Futa Tanaka ^4^, Takeshi Hara ^4,5^, Kazumasa Yoshii ^6^, Katsusuke Yamashita ^7^, Keisuke Nakano ^2^, Kiyofumi Takabatake ^2^, Hotaka Kawai ^2^_,_ Hitoshi Nagatsuka ^2^ and Yoshihiko Furuki ^1^

^1^ Department of Oral and Maxillofacial Surgery, Kagawa Prefectural Central Hospital, 1-2-1, Asahi-machi, Takamatsu, Kagawa, 760-8557, Japan, [gouwan19@gmail.com](mailto:gouwan19@gmail.com)

^2^ Department of Oral Pathology and Medicine, Okayama University Graduate School of Medicine, Dentistry and Pharmaceutical Sciences, Okayama, 700-8558, Japan

^3^ Department of Molecular Oral Medicine and Maxillofacial Surgery, Graduate School of Biomedical and Health Sciences, Hiroshima University, 1-2-3 Kasumi, Minami-ku, Hiroshima, 734-8553, Japan.

^4^ Department of Electrical, Electronic and Computer Engineering, Faculty of Engineering, Gifu University, 1-1 Yanagido, Gifu, Gifu, 501-1193, Japan

^5^ Center for Healthcare Information Technology, Tokai National Higher Education and Research System, 1-1 Yanagido, Gifu, Gifu 501-1193, Japan

^6^ Department of Intelligence Science and Engineering, Graduate School of Natural Science and Technology, Gifu University, 1-1 Yanagido, Gifu, Gifu, 501-1193, Japan

^7^ Polytechnic Center Kagawa, 2-4-3, Hananomiya-cho, Takamatsu, Kagawa 761-8063, Japan

***** Correspondence: gouwan19@gmail.com, Tel.: +81 87 811 3333, Fax: +81 87 835 8363

**Appendix**

***Performance metrics***

We evaluated the performance metrics, with precision, recall and the F1 score as defined in Equations 3–7, along with the receiver operating characteristic curve (ROC), and the area under the ROC curve (AUC). The ROC curves were shown for the complete dataset from the 10-fold cross-validation, producing the median AUC value.

In these equations, TP, TN, FP, and FN represent true positive (normal correctly identified), true negative (abnormal correctly identified), false positive (abnormal incorrectly identified), and false negative (normal incorrectly identified) results, respectively.

$accuracy=\frac{TP + TN}{TP + FP + TN + FN}$ (1)

$precision=\frac{\mathrm{TP}}{TP + FP}$ (2)

$recall=\frac{\mathrm{TP}}{TP + FN}$ (3)

$F1 score=2\times\frac{precision \times recall}{precision + recall}$ (4)

***Data augmentation***

Various data augmentation techniques were used in this study to prevent overfitting. During the training data learning, data augmentation was applied to the training image data when the images were taken out in batches. The training data were randomly zoomed out vertically in the 20% range and zoomed out horizontally in the 20% range.

***Figure S1: Average ROC of mandibular third molar classification by single-task and two types of multi-task learning.***

multi-3task; class, position and Winter’s classification

multi-2task; class and position

**
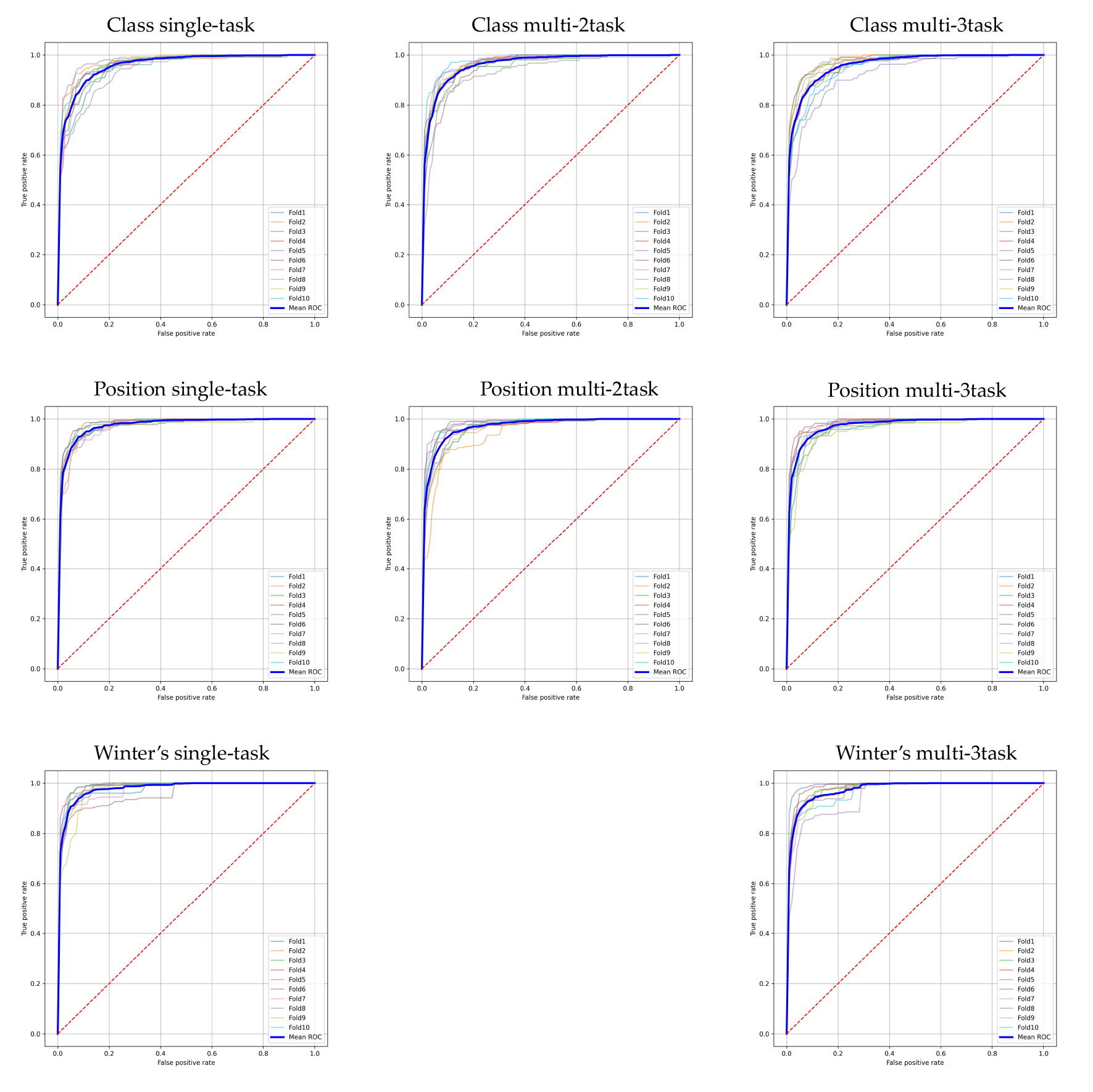
**

***Figure S2. Visualization of Model Classification***

Figure S2 shows ten representative images of mandibular third molars classified using each CNN model, visualized using Grad-CAM.

**
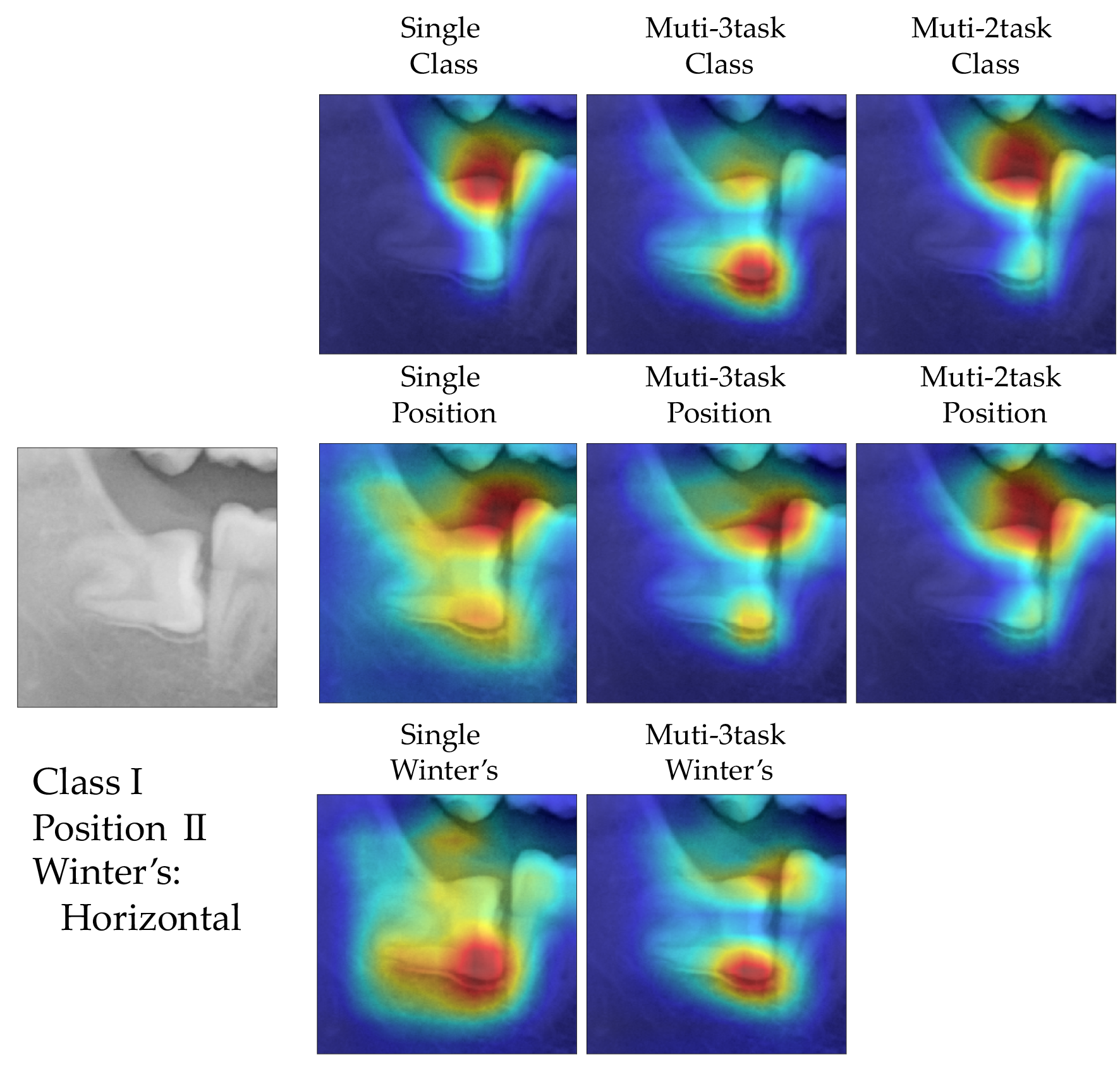
Winter’s classification; Holizontal**

**
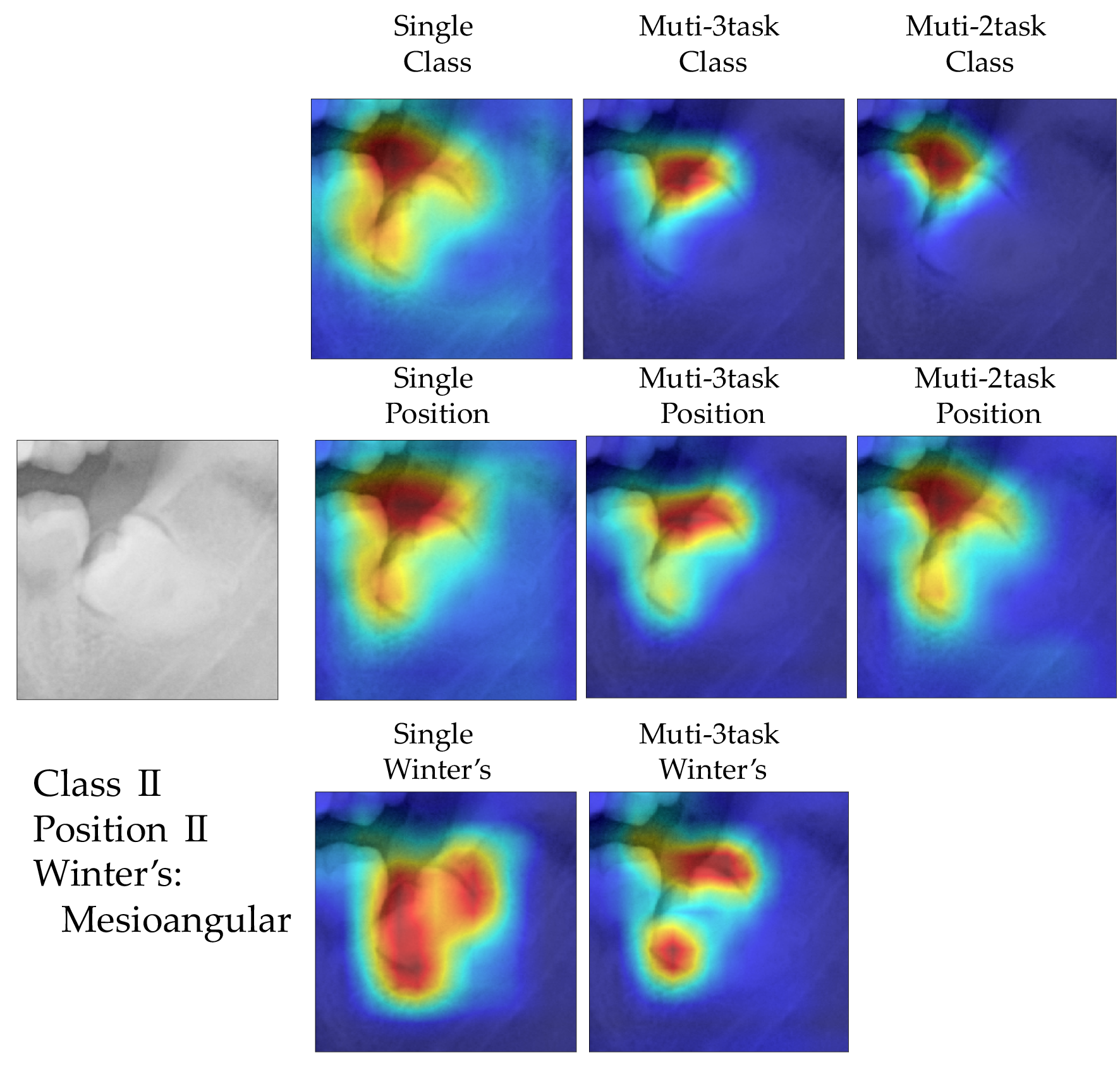
Winter’s classification; Mesioangular**

**Winter’s classification; Vertical**


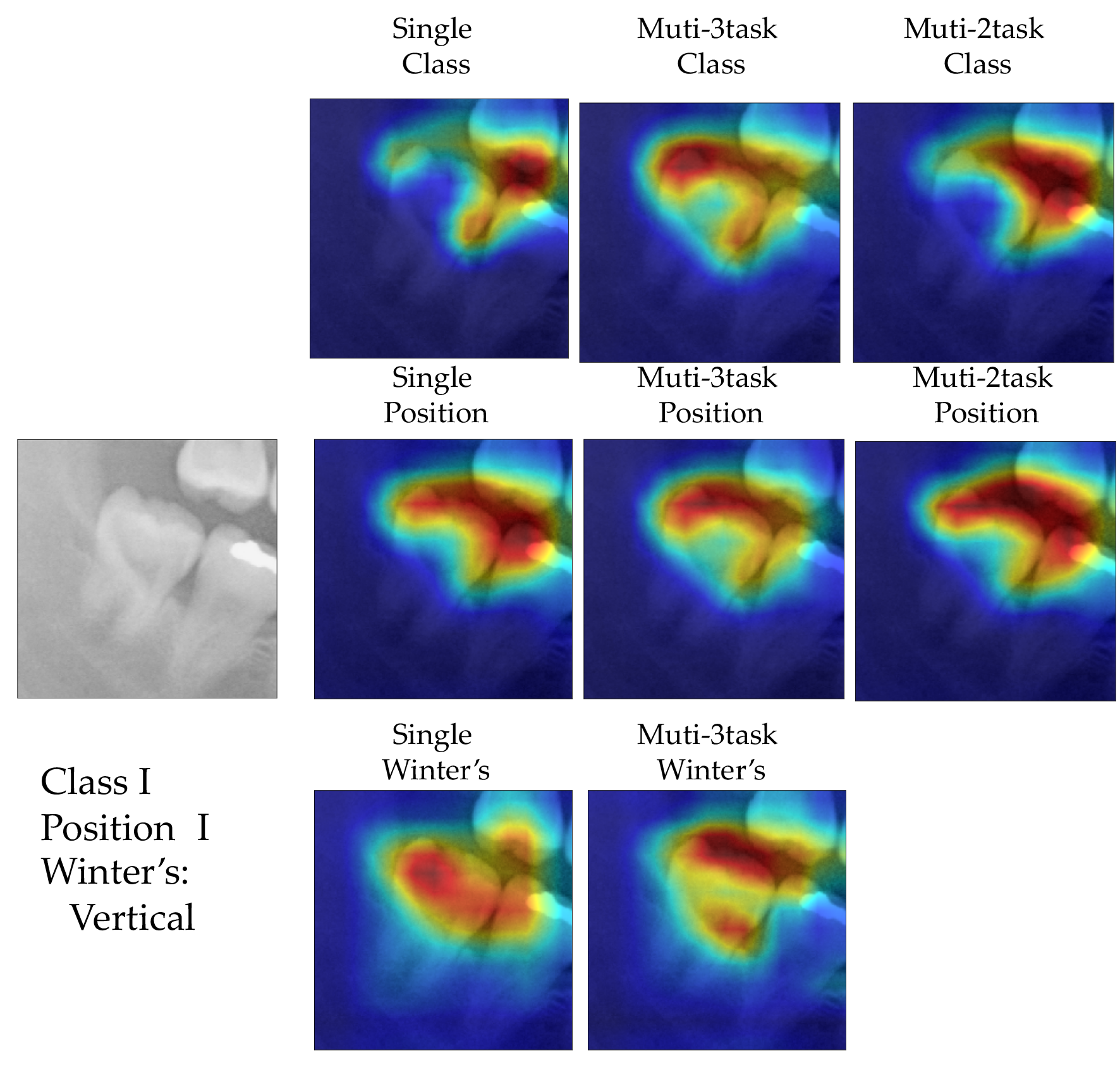


**Winter’s classification; Distoangular**


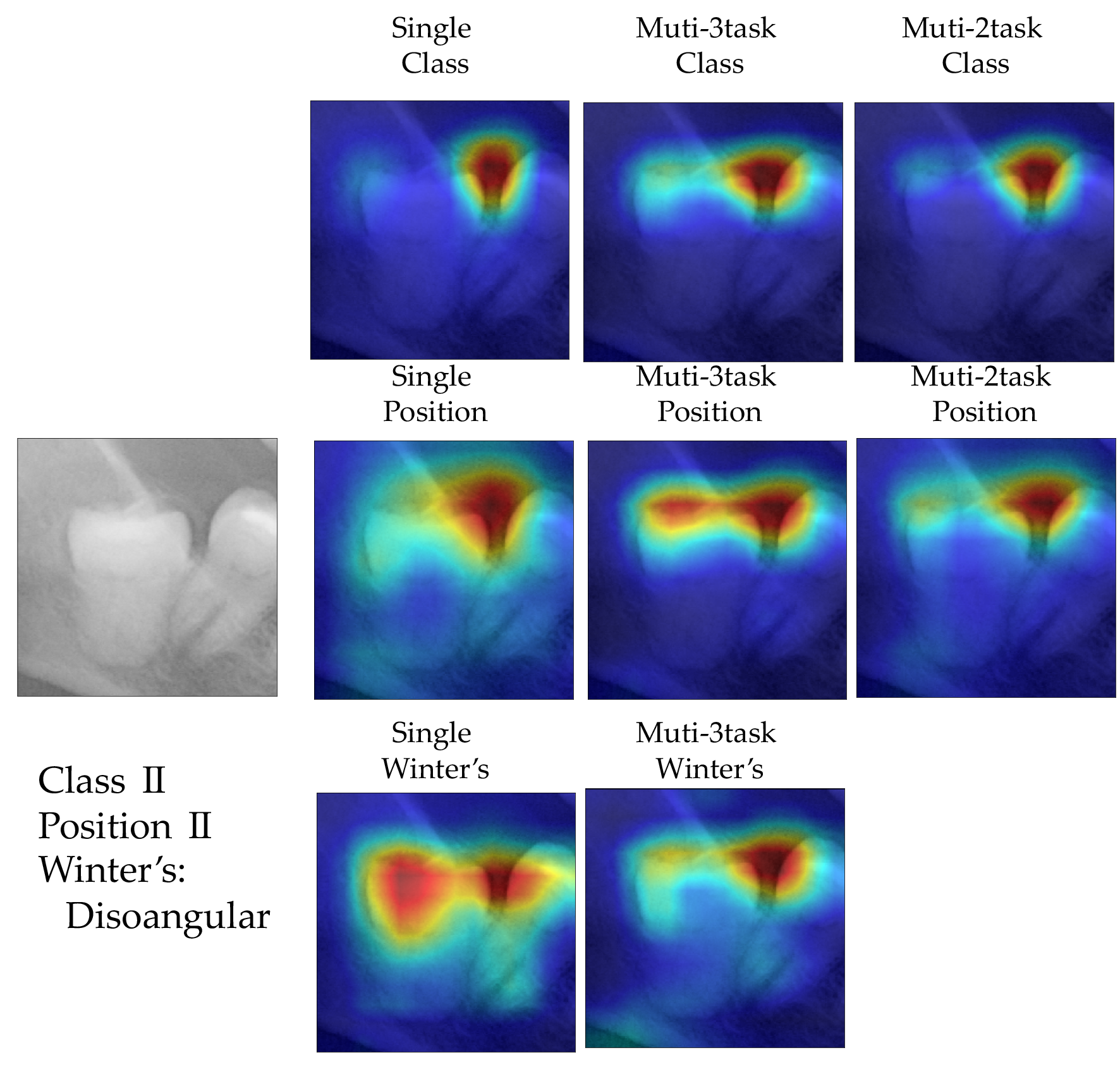


**Winter’s classification; Buccoangular or Lingualangular**


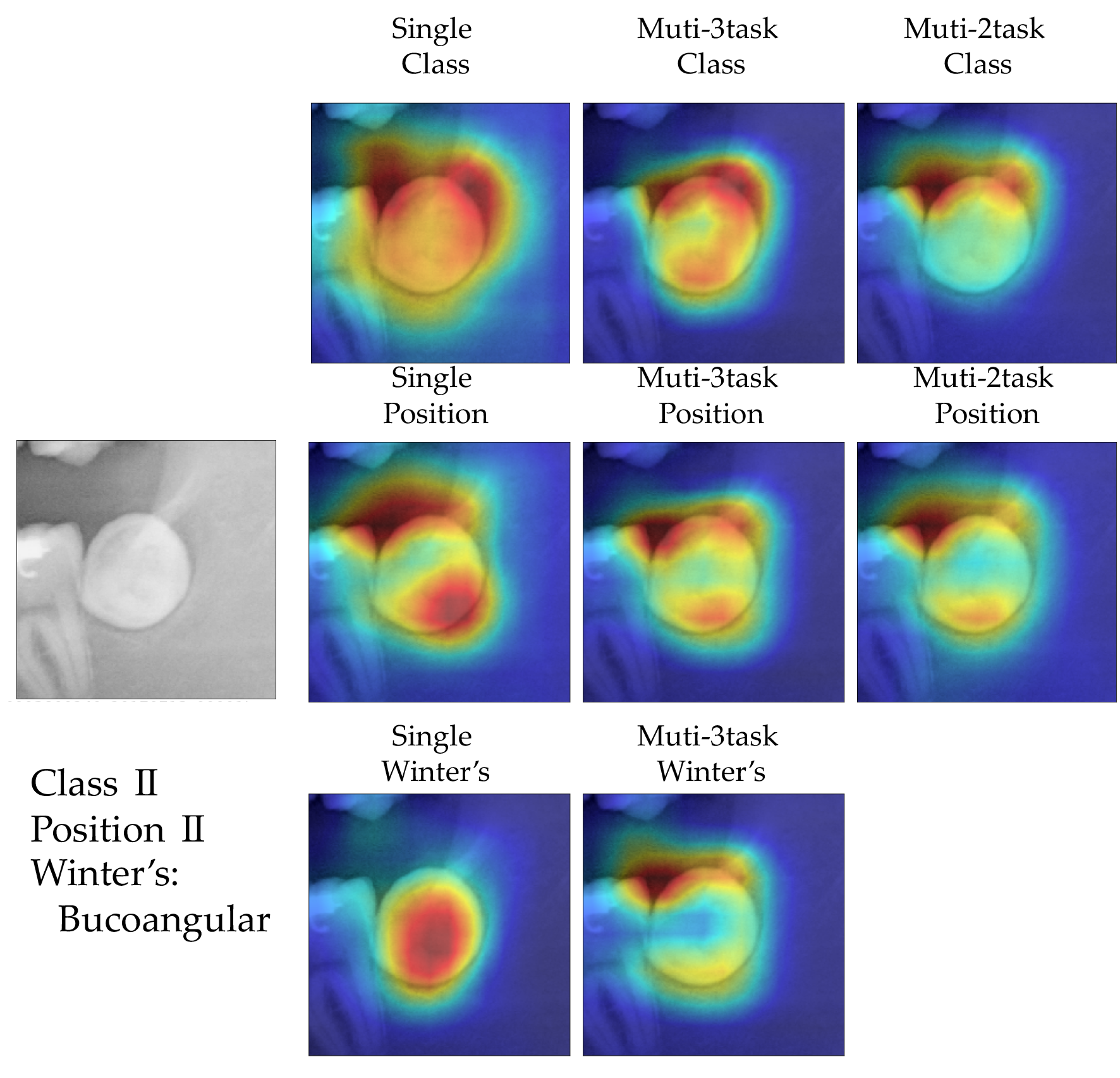


**Winter’s classification; Inverted**


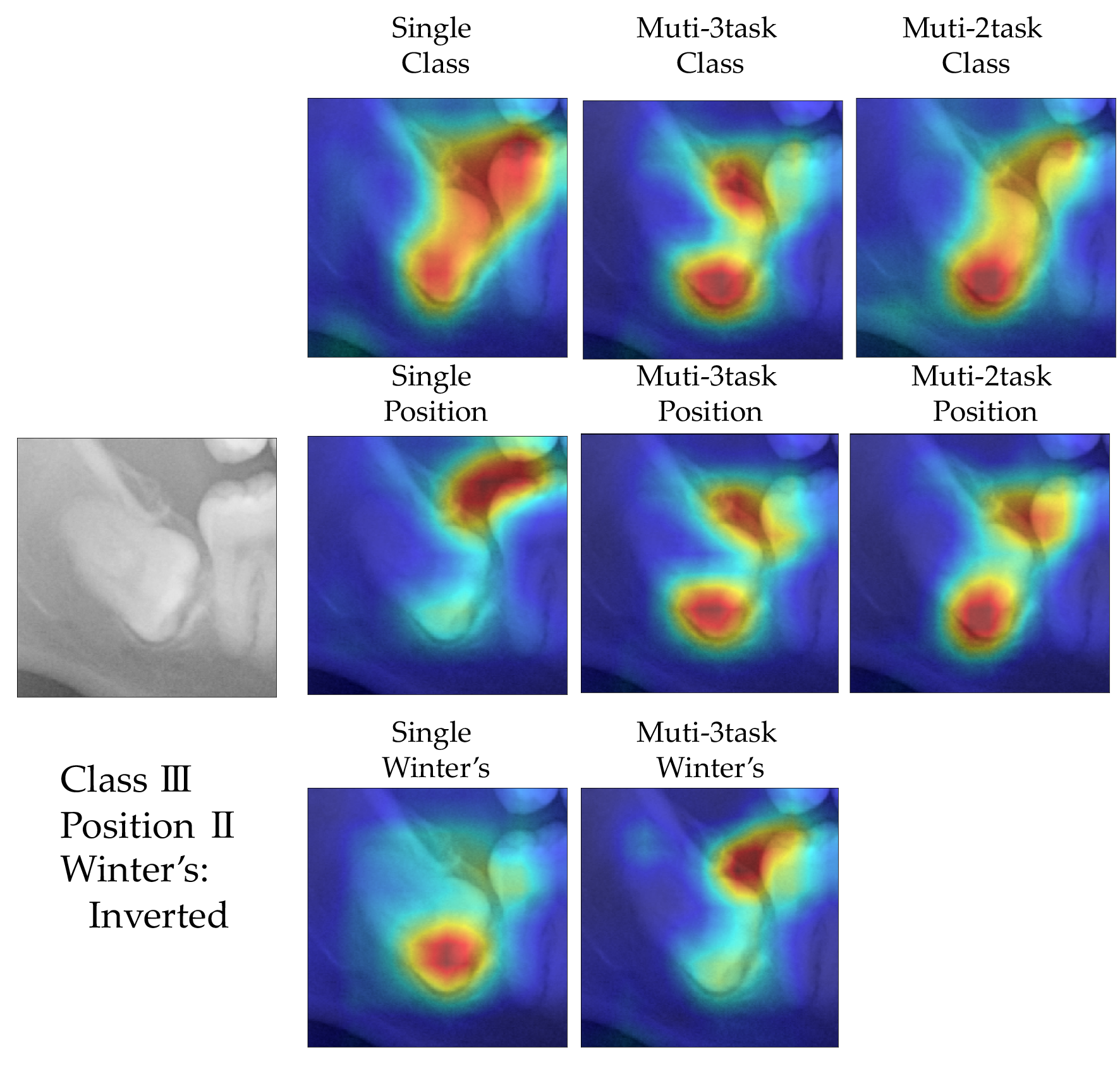

Supplement: Supplementary file 1 — Supplementary Information. [file 41598_2021_4603_MOESM1_ESM.docx]
